# Design of adaptive ensemble classifier for online sentiment analysis and opinion mining

**DOI:** 10.7717/peerj-cs.660

**Published:** 2021-08-05

**Authors:** Sanjeev Kumar, Ravendra Singh, Mohammad Zubair Khan, Abdulfattah Noorwali

**Affiliations:** 1 Department of Computer Science and Information Technology, M.J.P. Rohilkhand University, Bareilly, Uttar Pradesh, India; 2 Department of Computer Science, College of Computer Science and Engineering, Taibah University, Madinah, Madinah, Saudi Arabia; 3 Department of Electrical Engineering, Umm Al-Qura University, Makkah, Makkah, Saudi Arabia

**Keywords:** Sentiment analysis, Concept drift, Adaptive ensemble, Data stream mining, Drift-detection

## Abstract

DataStream mining is a challenging task for researchers because of the change in data distribution during classification, known as concept drift. Drift detection algorithms emphasize detecting the drift. The drift detection algorithm needs to be very sensitive to change in data distribution for detecting the maximum number of drifts in the data stream. But highly sensitive drift detectors lead to higher false-positive drift detections. This paper proposed a Drift Detection-based Adaptive Ensemble classifier for sentiment analysis and opinion mining, which uses these false-positive drift detections to benefit and minimize the negative impact of false-positive drift detection signals. The proposed method creates and adds a new classifier to the ensemble whenever a drift happens. A weighting mechanism is implemented, which provides weights to each classifier in the ensemble. The weight of the classifier decides the contribution of each classifier in the final classification results. The experiments are performed using different classification algorithms, and results are evaluated on the accuracy, precision, recall, and F1-measures. The proposed method is also compared with these state-of-the-art methods, OzaBaggingADWINClassifier, Accuracy Weighted Ensemble, Additive Expert Ensemble, Streaming Random Patches, and Adaptive Random Forest Classifier. The results show that the proposed method handles both true positive and false positive drifts efficiently.

## Introduction

Due to the enormous increase of data flowing on the internet, stream analysis has become an in-demand research area these days. Data are floating through in tremendous amounts on various social media platforms, online shopping, blogs, etc. Stream data analytics differ from offline or static data analysis. It has certain limitations, such as a single pass of data instance, less training data, massive data, concept drift, and imbalanced data. When we talk about review-based sentiment analysis, the scenario becomes more challenging as text data has high dimensions. Reviews are unpredictable, as people’s perception changes with time. For example, people always give thoughts about the government and its policies. Sometimes people have positive sentiments about government, and the data is imbalanced as most reviews are positive. If something happens that changes the people’s opinion about the government, then suddenly everything changes, and most reviews become negative. Classifier performance degrades in such a dynamic environment because of the shift in imbalance factor.

In a dynamic environment, the data distribution can change over time, and this phenomenon is known as concept drift (Janardan & Mehta, 2017; Kadam & Kadam, 2019). There can be two kinds of change in distribution. When the change happens in the conditional distribution of the output *P*(*y*_*t*_|*X*_*t*_),  *i.e.*, target variable, given the input, *i.e.*, features, while the input distribution may not change, which is called real concept drift. Another concept drift is called virtual concept drift, in which the change happens in the distribution of input variables $P \left( {X}_{t} \right) $ *i.e.*, features. Considering the concept of drift, the most complicated problem in stream-data analysis, various methods are proposed by researchers to deal with it. There are three types of strategies that are implemented:

• Drift detection-based methods
• Window-based methods
• Ensemble of methods

In drift detection-based concept drift handling procedures (Gama et al., 2004; Wang et al., 2013; Baena-garc et al., 2006; Wang & Abraham, 2015; Wares, Isaacs & Elyan, 2019), the algorithm considers various kinds of changes in performance during stream data analysis. DDM, EDDM, DDM-OCI, LFR, NFR, ADWIN, etc., are the algorithms that work on these principles. These algorithms focus on finding the accurate time when drift occurs. Another algorithm category is based upon the window of stream data (Du, Song & Jia, 2014). In these algorithms, the data stream is divided into a window of the same size. Algorithms try to find the change in performance in any window and then use these windows to retrain the classifiers. The problem with such algorithms is deciding the window’s size, as a big window will take a significant amount of time and memory to analyze, while a small size window can miss the concept drift between two windows. Another kind of algorithm is the ensemble of classifiers (Brzezinski & Stefanowski, 2014; Almeida et al., 2018; Van Rijn et al., 2016) to handle the concept drift. Researchers derive various kinds of methods from combining classifiers dynamically so that the concept drift doesn’t impact the performance. The ensemble classifier’s basic idea is to train and add a new classifier to the available ensemble whenever performance goes down. These methods based on ensemble classifiers primarily handle the concept drift without detecting it.

Researchers have also done some interesting work where different drift detection algorithms combine to ensemble drift detection algorithms (Maciel, Santos & Barros, 2016a; Du et al., 2015). The concept drift can be detected with fewer false-positive detections. Another problem with stream data analysis is imbalanced data. An imbalanced dataset has also occurred in a static dataset, but it is predefined and fixed in a static dataset: which one is a minor class and which one is a major class. In a dynamic dataset, *i.e.*, stream data, it is not fixed which class is minor and major. In-stream data, major and minor classes can be changed at various times. A minor class at the time *t*_1_ can become a major class at *t*_2_, which will create a problem for classification as it is trained in different settings. Imbalanced data can become balanced at some time and again change to an imbalanced dataset. So, the algorithms must deal with imbalanced data dynamically.

### Motivation and objectives

The real-world applications of sentiment analysis and opinion mining are the biggest motivation behind this research. Drift detection and drift handling are the biggest challenges in stream data analysis, which motivates further for this research work. Ensemble classifier has shown its efficiency in various applications. This encourages us to design an ensemble classifier for online sentiment analysis and drift handling.

The key issues this study has addressed are sentiment analysis and opinion mining of text review data stream, drift detection and handling in text data stream, design of an ensemble classifier for improving the accuracy of online sentiment analysis and opinion mining. The novelty of this work is to improve performance for sentiment analysis and opinion mining on text review data stream with drift detection and drift handling by designing an ensemble classifier. False positive drift detection is a common problem with most of the drift detection algorithm. The Linear Four Rates drift-detection algorithm is used in this paper for accurate drift detection. This algorithm also has the problem of false positive drift detection and the performance of a classification method degrades due to this. In this novel proposed method, an ensemble classifier will be created to improve the performance for sentiment analysis.

The objectives of this study are:

1. To design of a Drift detection based Adaptive Ensemble Classifier for online sentiment analysis and opinion mining.
2. The ensemble classifier should handle true positive and false positive both kind of drift detection signals.
3. To evaluate the technique using well known parameters and comparing the performance with other state-of-the-art algorithms of stream mining.
4. To validate the significance of the results obtained using a statistical method.

The rest of the paper is organized as follows. Section 2 gives the background of techniques used and the work done related to data stream mining and ensemble classifiers. Section 3 explains the proposed Drift-detection-based Adaptive Ensemble (DAE) model. Section 4 describes the experimental work and results. The discussion related to the experimental result is in Section 5. Section 6 is the conclusion and future work.

## Background and related work

Du, Song & Jia (2014) used a sliding window for determining the concept drift. Determining the size of the window is a complicated task in the sliding window technique. Gama et al. (2004) proposed a method that controls algorithms’ error rate to determine the concept drift. This method is known as DDM (Drift Detection Method), which considers *P*_*i*_ the error rate and *S*_*i*_ standard deviation, two variables that determine the change in distribution. DDM does not perform well on an imbalanced dataset. Wang et al. (2013) proposed DDM-OCI (Drift Detection Method-Online Class Imbalance), which considers minority class recall for capturing the drift. This method performs very well with the imbalanced data stream. This method needs to know the majority and minority classes in advance, which is a limitation of this algorithm. Baena-garc et al. (2006) proposed EDDM (Early Drift Detection Method), which considers the distribution of the distance between classifier errors. The work claims that EDDM detects change faster with fewer false-positive detections.

Wang & Abraham (2015) proposed an algorithm for drift detection named Linear Four Rates (LFR), which considers four parameters, true positive rates (TPR), true negative rates (TNR), positive predictive value (PPV), and negative predictive value (NPV) for detecting concept drift. This algorithm can handle imbalanced data when the majority and minority classes are not known in advance. Yu et al. (2019) implemented Hierarchical Linear Four Rates (HLFR) under the hierarchical hypothesis testing framework. This algorithm minimizes the false positive drift detection by working in two layers. The first layer implements LFR and detects the concept drift. The second layer performs a permutation test to confirm if the concept drift is true positive or not. Sun, Shao & Wang (2019) proposed a change detection model for a different type of concept drift detection based on the Jensen–Shannon divergence. This method considers label dependency by pruning away infrequent label combinations to enhance classification performance.

Maciel, Santos & Barros (2016b) combine three drift detectors for accurate concept drift detection. This mechanism minimizes the false positive drift as a drift must be recognized by most drift detectors in the drift detector ensemble. Du et al. (2015) proposed a selective detector ensemble for abrupt and gradual drift detection. The proposed algorithm combines the base detectors during detections dynamically using the early-find-early-report fusion rule to discover concept drift. The above-discussed works are some of the recent works done for concept drift detection in a data stream. Another approach for handling concept drift is using an ensemble of classifiers in which a stream classification model improvises itself with classification, sometimes detecting concept drift separately and sometimes not. Brzeziński & Stefanowski (2011) extended the weighted accuracy ensemble using an online component classifier and updated them according to the current distribution. In another paper, Brzezinski & Stefanowski (2014) proposed a new weighted and updated mechanism and made some other changes to reduce computational costs and improve classification accuracy.

De Almeida et al. (2017) proposed a framework that can keep several classifiers in a pool trained on different data sets at different times and dynamically select a classifier for the ensemble from this pool for an instance classification. In another paper, Almeida et al. (2018) introduced the concept of diversity according to which a classifier trained under different concepts may be beneficial for concept drift handling. Van Rijn et al. (2016) proposed an ensemble classifier with heterogeneous base classifiers. This paper presents an online performance estimation framework, which can be used to weight the classifier’s votes in an ensemble. Katakis, Tsoumakas & Vlahavas (2008) proposed a framework that designs ensemble classifiers, especially for recurring concept drift handling. This framework exploits incremental clustering to build an ensemble dynamically and update the ensemble of incremental classifiers. The ensemble is produced by maintaining a classifier for each concept. Minku & Yao (2012) focus on diversity between classifiers to create an ensemble that could attain better accuracy than other approaches. This paper used two ensembles, one with lower diversity and one with higher diversity. It used a drift detection method to detect drift. Both ensembles were trained on upcoming data instances. Before detecting drift, it used a lower diversity ensemble, but it used a higher diversity ensemble for classification after drift detection. Cano & Krawczyk (2020) proposed an ensemble model that uses Kappa statistics for dynamic weighting and the selection of base classifiers. This is a combination of the online and block-based ensemble. Zhang et al. (2017) proposed a three-layer model for concept drift detection for text data streams. In this model, the three layers represent the layer of label space, the layer of feature space, and the mapping relationships between labels and features.

Drift detection algorithms such as DDM, EDDM, LFR, etc., can only detect the drift and provide the drift’s location and severity, but these algorithms are not for drift handling. The classifier is used for prediction updates whenever a drift occurs to handle the drift. It has been proven that the ensemble of classifiers performs well compared to a single classifier on static datasets. In dynamic datasets or stream analysis, classifiers’ ensemble could be beneficial with drift detection because the ensemble of classifiers provides flexibility to handle different types of concept drifts. The drift detection algorithm provides the drift’s location, width, and severity. Khamassi et al. (2013) did this by combining ensembles such as online bagging and online boosting with the drift detection method EDIST. EDIST is an error distance-based method for drift detection based on EDDM. Khamassi combines the EDIST with online bagging and online boosting. EDIST detects the drift, and these ensemble methods create an ensemble with the Hoeffding Tree to cope with the drift and preserve the performance after the occurrence of drift. In this method, the problem with EDIST is that it is based on EDDM, and both detect drift using the distance between error, which does not work very well with imbalanced datasets.

Li, Xiong & Huang (2019) combined the DDM method for drift detection with an incremental ensemble for drift handling with the same idea of combining drift detection algorithms with ensemble classifiers. Drift-detection-based incremental ensemble updates the weight on ensemble trees. An alternate version of the Hoeffding adaptive tree replaces the old tree, enhancing the model’s ability to deal with sudden drift. In this method, all other ensemble members are also updated with the latest data arrived. This method performs well with standard data streams, but it has a weakness, as this method uses DDM for drift detection, which is an error-based drift detection method and cannot work well when the data stream contains imbalanced data.

Chauhan, Sharma & Sikka (2021) surveyed the election results prediction using sentiment analysis on social media content. The author found out that most of the research work used twitter as their data source to collect data for sentiment analysis. The outcomes of this survey were based on 38 research papers. In most of the research works, lexicon-based techniques were used for sentiment analysis. Out of these 38 research articles, only three to four articles used the machine learning technique for sentiment analysis. This work also discussed research challenges in the field of sentiment analysis for election prediction.

Jain & Jain (2021) presented a hybrid feature selection technique for sentiment classification. The author combined Information Gain, CHI Square, and GINI Index to find the feature set and then reduces this feature set using UNION SET operation. The Genetic Algorithm was used for the optimization of these feature sets. The four different variants of SVM were used for sentiment classification using the features obtained by the Genetic Algorithm after optimization.

Ray, Garain & Sarkar (2021) proposed a hotel recommendation system using sentiment analysis of hotel reviews. First, an ensemble of the BERT model was used to find the word embeddings. Then these pre-trained word embeddings were fed to the Random Forest Classifier with other textual features such as word vectors, TF-IDF of frequent words, subjectivity score. The author also categorized the dataset based on aspects using fuzzy logic and cosine similarity.

A summary of some of the important works done in-stream data analysis is shown in Table 1.

**Table 1:** The summary of related work done in stream data analysis.

**Sr. no**	**Author (Year)**	**Title**	**Topic/Focus/Purpose**	**Paradigm and method**	**Pros or cons**
1	L. Du, Q. Song, and X. Jia (2014)	Detecting concept drift: An information entropy-based method using an adaptive sliding window	Window Based Drift Detection Algorithm	Sliding Window Technique for concept drift detection, Window size determined dynamically, entropy is used for drift detection	Cons: determination of window size is difficult
2	J. Gama, P. Medas, G. Castillo, and P. Rodrigues (2014)	Learning with drift detection	Statistical (Error rate based) drift detection method	DDM, used error rate for drift detection, change in error rate determines the change in distribution,	Cons: problems with imbalanced data
3	S. Wang, L. L. Minku, D. Ghezzi, D. Caltabiano, P. Tino, and X. Yao (2013)	Concept drift detection for online class imbalance learning	Statistical (minority class recall-based) Drift detection method	DDM-OCI, considers minority class recall for drift detection, works well with imbalanced data,	Pros: good with imbalanced data, cons: minority and majority class should be known already
4	Heng Wang and Zubin Abraham (2015)	Concept drift detection for streaming data	Statistical (Linear four rates) Drift detection method	Linear Four Rates (LFR) considers tpr, tnr, ppv and npv for drift detection,	Pros: works good with imbalanced data, works fine when imbalance data changes in balance data, cons: Problem of false positive, very sensitive to any change in data distribution and noise
5	Shujian Yu et al. (2019)	Concept drift detection and adaptation with hierarchical hypothesis testing	Two-layer hypothesis test-based drift detection method	upgraded Linear Four Rates to Hierarchical Linear Four Rates, using two-layer architecture tries to minimize the false positive	Pros: minimizes the false positive drift detection Cons: multiple tests each time
6	Dariusz Brzezinski and Jerzy Stefanowski (2011)	Accuracy updated ensemble for data streams with concept drift	Accuracy Based ensemble classifier for drift handling	Accuracy Updated Ensemble, select classifier with Hoeffding Option Tree, update classifiers with current distribution,	Cons: Limited diversity between classifiers in ensemble
7	Iman Khamassi et al. (2013)	Ensemble classifiers for drift detection and monitoring in dynamic environments	Ensemble classifier for concept drift detection and monitoring	Combines online bagging and boosting with EDIST drift detection algorithm,	Cons: EDIST is an error distance-based algorithm, doesn’t work well with imbalanced data
8	Zeng Li et al. (2019)	Drift-detection Based Incremental Ensemble for Reacting to Different Kinds of Concept Drift	Drift detection-based ensemble classifier	Combines DDM, a drift detection method with an incremental ensemble. Works fine with sudden drift.	Cons: DDM is an error-based method, doesn’t work well with imbalanced data.

Finally, Wang & Abraham (2015) have proposed a drift detection algorithm Linear Four Rates which can detect drift in the imbalance dataset very efficiently. The four rates used in this algorithm to detect the drift are very sensitive metrics, and because of that, LFR can detect any concept drift. But this sensitivity of these four rates become the reason for triggering more false-positive detections. Yu et al. (2019) tried to minimize the false positive detections using the permutation test. In our proposed hypothesis, instead of reducing the false positive detection, we can reduce the effect of false-positive detection using an ensemble of classifiers. The next section shows the detailed implementation of drift detection based adaptive ensemble.

## Drift-Detection based Adaptive Ensemble (DAE)

In DAE, we proposed the combination of linear four rates for drift detection and an adaptive ensemble that adds classifiers when LFR detects drift. LFR established itself as one of the best drift detection algorithms. It can handle those imbalanced datasets in which minority and majority classes are not fixed in advance and change during online classification. In the LFR drift detection algorithm, the hypothesis testing is done by estimating the rates in *P*_∗_ at each time step. The statistical hypothesis testing is done at each time step t using the following null and alternative hypotheses.

\begin{eqnarray*}{H}_{0}:\forall \ast ,P \left( estimator~of~{P}_{\ast }^{ \left( t \right) } \right) =P \left( estimator~of~{P}_{\ast }^{ \left( t-1 \right) } \right) \end{eqnarray*}

\begin{eqnarray*}{H}_{a}:\exists \ast ,P \left( estimator~of~{P}_{\ast }^{ \left( t \right) } \right) \not = P \left( estimator~of~{P}_{\ast }^{ \left( t-1 \right) } \right) \end{eqnarray*}

If no concept drift is detected, and the concept is stable under the null hypothesis; if the null hypothesis is rejected, the concept drift is detected. The LFR drift detection mechanisms compare the significance level with two user-defined significance levels under the null hypothesis *H*_0_. The two user-defined significance levels are warning level (*δ*_∗_) and detect level (*ϵ*_∗_). The type 1 error occurs when there is no drift, but the drift detection algorithm signals a new drift. This is called false positive drift detection. A type 2 error occurs when there is a drift happening, but the drift detection algorithm detects nothing. This is called false-negative drift detection. Each drift detection algorithm focuses on minimizing the type 2 error, which means detecting every drift happening in the data stream. While focusing on detecting every possible drift in the dataset, the drift detection algorithm becomes very sensitive to any change in the data stream, increasing type 1 error, which means false positive detection. For each detected drift, the algorithm needs to relearn. In the case of false positive drift detection, it results in classifier performance degradation. This paper proposed a drift detection-based adaptive ensemble classifier for classification to minimize false-positive drift detection’s side effects. The proposed model hypothesis is based on the concept that an ensemble classifier contains multiple classifiers that can learn the new pattern while retaining the previously known patterns.

**Figure 1:**
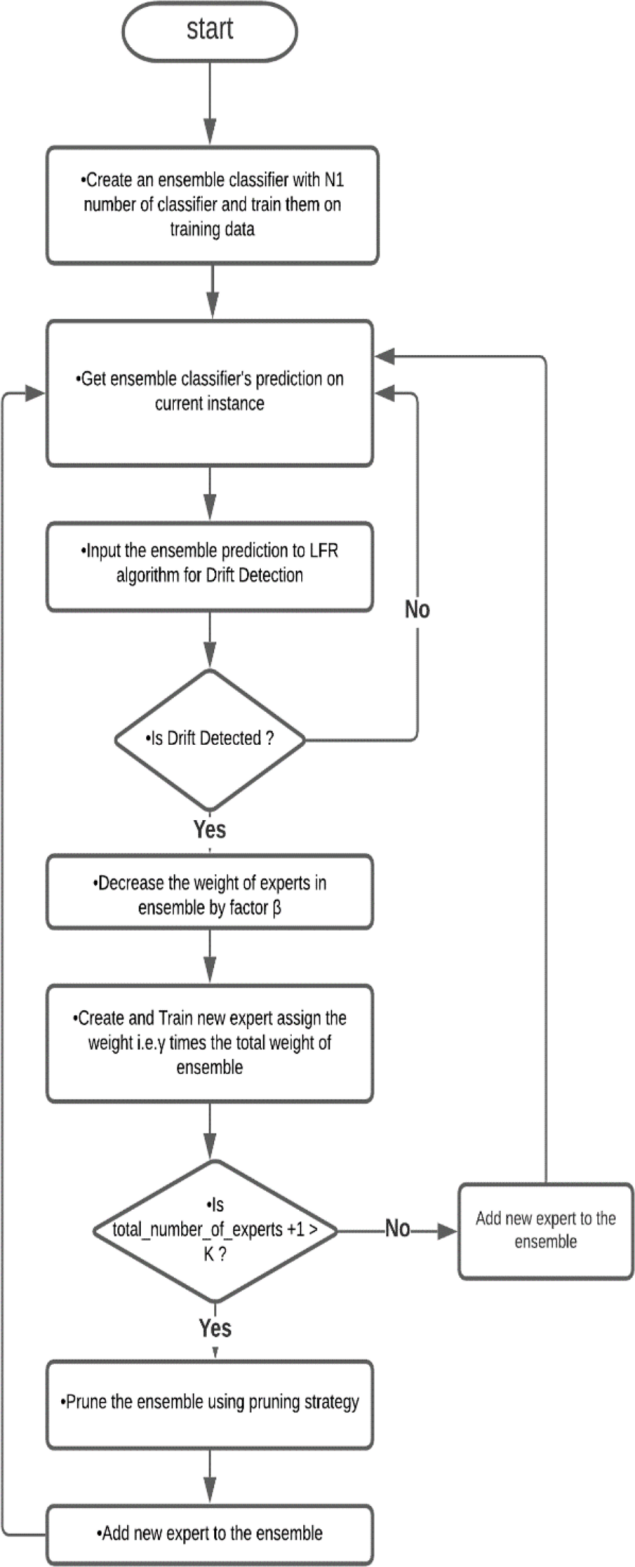
Flowchart of Drift-detection based Adaptive Ensemble Model. This is a flow chart of the Drift detection-based Adaptive Ensemble, which shows the flow of algorithms.

In this method, first, we prepared an ensemble with a minimum number of classifiers; in this paper, we used five classifiers and trained them on a training dataset. The ensemble classified an instance and provided the predicted result in the next step. Using the actual result and predicted outcome, the LFR algorithm works and checks for any drift. If no drift is detected, the process repeats for another instance. If LFR stated that drift is detected, then a new classifier trained on the dataset is achieved from a warning to detection instances. This new classifier is added to the ensemble, and the weight of the classifier and ensemble are updated by the adaptive ensemble’s weight management algorithm. The flowchart of the algorithm is shown in Fig. 1.

## Linear four rates for drift detection

As a drift detection mechanism, LFR has several advantages, such as working for different types of concept drifts. It can handle imbalanced data very well for concept drift detection. Wang & Abraham (2015) established that LFR could detect concept drift earlier than other existing approaches such as DDM or DDM-OCI. LFR can detect concept drift with the least false alarms and has the best recall comparing different drift detection approaches. Another important property of LFR is that this algorithm is not dependent upon the underlying classifier employed.

Detecting concept drift is the detection of change point *P*(*X*_*t*_, *y*_*t*_). LFR detects change point in *P*(*f*(*X*_*t*_), *y*_*t*_), where *f* is the classifier for prediction. LFR uses the fact that any drift of *P*(*f*(*X*_*t*_), *y*_*t*_) would imply a drift in *P*(*X*_*t*_, *y*_*t*_) with a probability of 1. If there is a data stream (*X*_*t*_, *y*_*t*_) and $f \left( {X}_{t} \right) ={\hat {y}}_{t}$ is a binary classifier. The confusion probability matrix for classifier *f* is shown in Table 2.

**Table 2:** Confusion matrix for binary classification.

**Predicted\Actual**	**0**	**1**
**0**	TN	FN
**1**	FP	TP

Now the LFR uses four characteristic rates to detect the change in *P*(*f*(*X*_*t*_), *y*_*t*_), given as:

$True~Positive~Rate~{P}_{tpr}= \frac{TP}{TP+FN} $ ,

$True~Negative~Rate~{P}_{tnr}= \frac{TN}{TN+FP} $ ,

$Positive~Predicted~Value~{P}_{ppv}= \frac{TP}{TP+FP} $ ,

$Negative~Predicted~Value~{P}_{npv}= \frac{TN}{TN+FN} $ .

If the concepts remain stable, these four characteristic rates *P*_*tpr*_, *P*_*tnr*_, *P*_*ppv*_, *P*_*npv*_ will not change, but if any significant amount of change is detected in any of the four characteristic rates *P*_∗_, where $\ast \in \left\{ tpr,tnr,ppv,npv \right\} $, it represents a change in the underlying joint distribution (*f*(*X*_*t*_), *y*_*t*_) or concept.

LFR conducts the statistical test at each time step *t*, called ‘continuing test.’ This drift detection algorithm uses a linear combination of the classifier’s previous performance and current performance as the test statistic represented as ${R}_{\ast }^{ \left( t \right) }$ where $\ast \in \left\{ tpr,tnr,ppv,npv \right\} $ at each time step *t*. This equation updates as:

(1) \begin{eqnarray*}{R}_{\mathrm{\ast }}^{ \left( t \right) }\leftarrow {\eta }_{\mathrm{ \ast }}{R}_{\mathrm{\ast }}^{ \left( t-1 \right) }+ \left( 1-{\eta }_{\mathrm{ \ast }} \right) {1}_{\{ {y}_{t}={\hat {y}}_{t}\} }\end{eqnarray*}

where, *η*_∗_ is a time decay factor.

LFR uses the time decaying factor (*η*_∗_), warning significance level (*δ*_∗_),  and detect significance level (*ϵ*_∗_) as user-defined parameters for each rate in ∗.*η*_∗_ is used to evaluate the classifier’s performance of current instance prediction while *δ*_∗_ and *ϵ*_∗_ are the statistical significance levels for warning and drift detection. Using these significance levels with the Monte Carlo Simulation, warning and drift detection limits can be calculated as *warn*.*bd*_∗_ and *detect*.*bd*_∗_. During the online classification, if any ${R}_{\ast }^{ \left( t \right) }$ goes beyond the *warn*.*bd*_∗_ for the first time, it is considered that a concept drift is likely to occur, and *warn*.*time* is set to *t*. After that, if any ${R}_{\ast }^{ \left( t \right) }$ goes beyond *detect*.*bd*_∗_, concept drift is confirmed, and *detect*.*time* is set at *t*. All instances extracted from *warn*.*time* to *detect*.*time* are used to update the ensemble classifier.

## Adaptive ensemble for classification and prediction

An ensemble of predictive models was maintained to classify and predict data stream instances in the DAE. The predictive models in this ensemble are referred to as experts. All experts use the same algorithm for training and prediction, but experts are trained on different time steps with varying data instances for obtaining the diversity between experts. The weighted vote of each expert determines the performance of experts. The algorithm adds all the experts’ predicting that class weight and the class with maximum weight is selected as the final result for each class. After prediction, LFR processes the outcome and determines that drift is detected. When LFR signals that drift is detected, the weight of all the experts is decreased by multiplicative factor *β*. After that, a new expert is trained on the data instances extracted from the data stream between time steps *warn*.*time* and *detect*.*time*. Finally, this new expert is added to the ensemble. The weight of the newly added classifier will be equal to the total weight of the ensemble and multiplied by constant *γ*. Here we can also train other experts in ensemble with the data instances that arrived between *warn*.*time* and *detect*.*time*. We can compare the result when all experts have adaptively trained and when only the newly added expert is trained.

**Algorithm: Drift-Detection Based Adaptive Ensemble (DAE)**

$\mathbi{Input}:Data\{ \left( {X}_{t},{y}_{t} \right) \} _{t=1}^{\mathrm{\infty }}where~{X}_{t}\in {R}^{d}~and~{y}_{t}\in \{ 0,1\} $

***Parameters***:

$\beta \in \left[ 0,1 \right] :for~decreasing~weight~of~ensemble~elements$

$\gamma \in \left[ 0.1 \right] :Factor~for~New~Expert~Weight$

${\eta }_{\mathrm{\ast }}\in \left[ 0,1 \right] :Time~Decaying~factor$

${\delta }_{\mathrm{\ast }}\in \left[ 0,1 \right] :Warning~Significance~Level$

${\epsilon }_{\mathrm{\ast }}\in \left[ 0,1 \right] :Detect~Significance~Level$

*K*:*Maximum* *number* *of* *experts* *ensemble* *can* *contain*

***Initialization***:

1. *Set* *of* *initial* *number* *of* *expert* *N*_1_←5
2. *Set* *of* *initial* *expert* *weight* *w*_1,1_←1
3. ${P}_{\mathrm{\ast }}^{ \left( t \right) }\leftarrow 0.5,{R}_{\mathrm{\ast }}^{ \left( t \right) }\leftarrow 0.5~where~\mathrm{ \ast }\hspace*{2.22198pt}\in \{ tpr,tnr,ppv,npv\} $
4. $Confusion~Matrix~{C}^{ \left( 0 \right) }\leftarrow \left[ 1,1;1,1 \right] $
5. *for* *t* = 1 *to* ∞ do:
6. *Get* *expert* *prediction* *E*_*t*,1_, …, *E*_*t*,*N*_*t*__ ∈ *Y*
7. Output Prediction ŷ_t_$\leftarrow argma{x}_{c\in Y}{\mathop{\sum }\nolimits }_{i=1}^{{N}_{t}}{w}_{t,i}[c={E}_{t,i}]$
8. *C*^(*t*)^ [ ŷ_t_ ] $ \left[ {y}_{t} \right] \leftarrow {C}^{ \left( t \right) }$ [ ŷ_t_ ] $ \left[ {y}_{t} \right] +1$
9. $for~each~\ast \hspace*{2.22198pt}\epsilon \left\{ tpr,tnr,ppv,npv \right\} do$
10. $if\ast ~influenced~by({y}_{t,}{\hat {y}}_{t})$ :
11. ${R}_{\ast }^{ \left( t \right) }\leftarrow {\eta }_{\ast }{R}_{\ast }^{ \left( t-1 \right) }+ \left( 1-{\eta }_{\ast } \right) {1}_{\{ {y}_{t}={\hat {y}}_{t}\} }$
12. *else*
13. ${R}_{\mathrm{\ast }}^{ \left( t \right) }\leftarrow {R}_{\mathrm{\ast }}^{ \left( t-1 \right) }$
14. *end* *if*
15. if $ \left( \ast \hspace*{2.22198pt}\in \left\{ tpr,tnr \right\} \right) :$
16. ${N}_{\ast }\leftarrow {C}^{ \left( t \right) } \left[ 0,{1}_{ \left\{ \ast =tpr \right\} } \right] +{C}^{ \left( t \right) }[1,{1}_{ \left\{ \ast =tpr \right\} }]$
17. ${P}_{\mathrm{\ast }}^{ \left( t \right) }\leftarrow \frac{{C}^{ \left( t \right) } \left[ {1}_{ \left\{ \ast =tpr \right\} },{1}_{ \left\{ \ast =tpr \right\} } \right] }{{N}_{\ast }} $
18. *else*
19. $\hspace*{20.00003pt}{N}_{\ast }\leftarrow {C}^{ \left( t \right) } \left[ {1}_{ \left\{ \ast =ppv \right\} },0 \right] +{C}^{ \left( t \right) } \left[ {1}_{ \left\{ \ast =ppv \right\} },1 \right] $
20.   ${P}_{\ast }^{ \left( t \right) }\leftarrow \frac{{C}^{ \left( t \right) } \left[ {1}_{ \left\{ \ast =ppv \right\} },{1}_{ \left\{ \ast =ppv \right\} } \right] }{{N}_{\ast }} $
21. *end* *if*
22. $warn.b{d}_{\ast }\leftarrow BoundTable \left( {P}_{\ast }^{ \left( t \right) },{\eta }_{\ast },{\delta }_{\ast },{N}_{\ast } \right) $
23. $detect.b{d}_{\ast }\leftarrow BoundTable \left( {P}_{\ast }^{ \left( t \right) },{\eta }_{\ast },{\varepsilon }_{\ast },{N}_{\ast } \right) $
24. *end* *for*
25. $if \left( any~{R}_{\ast }^{ \left( t \right) }exceeds~warn.b{d}_{\ast }\mathrm{{\XMLAMP}}warn.time=0 \right) :$
26. *warn*.*time*←*t*
27. $else~if \left( no~{R}_{\ast }^{ \left( t \right) }exceeds~warn.b{d}_{\ast } \right) then$
28. *warn*.*time*←0
29. *end* *if*
30. $if \left( any~{R}_{\ast }^{ \left( t \right) }exceeds~detect.b{d}_{\ast } \right) :$
31. *detect*.*time*←*t*
32. *Update* *expert* *weights* *w*_*t*+1,*i*_←*w*_*t*,*i*_*β*
33. *Add* *new* *expert* *N*_*t*+1_←*N*_*t*_ + 1
34. ${w}_{t+1,{N}_{t+1}}\leftarrow \gamma {\mathop{\sum }\nolimits }_{i=1}^{{N}_{t}}{w}_{t,i}$
35. $if \left( {N}_{t+1}\gt K \right) :$
36. *remove* *an* *expert* *based* *on* *pruning* *technique*
37. *end* *if*
38. *Train* *each* *expert* *in* *examples* *extracted*
39. *between* *warn*.*time* *and* *detect*.*time*
40. $reset{R}_{\ast }^{ \left( t \right) },{P}_{\ast }^{ \left( t \right) },{C}^{ \left( t \right) }as~step~1$
41. *end* *if*
42. *end* *for*

## Pruning strategies

With each drift occurrence, a new expert is added to the ensemble. If the drift frequently occurs, many experts may be added to the ensemble. As the drift occurs and the concept changes frequently, the previously added classifiers lose their significance and contribute very little to the ensemble’s final result. In this scenario, it was recommended that some classifiers needed to be pruned from the ensemble classifier. In the DAE, the maximum number of experts *K* allowed in the ensemble was defined previously. Whenever a new classifier was added to the ensemble, it was checked that the total number of experts did not exceed the maximum number of experts *K*allowed in the ensemble. If the total number of experts exceeded the limit, a pruning strategy was used to discard an expert from the ensemble. There are two pruning strategies available to use.

### Weakest first pruning method

When adding a new expert, if the total number of experts in the ensemble exceeds the maximum limit *K*, then the expert with the least weight is removed from the ensemble before adding a new expert.

### Oldest first pruning method

After adding a new classifier, if the total number of experts exceeds the maximum number of experts allowed *K*, then the oldest expert is removed from the ensemble, assuming that the oldest expert represents the oldest concept and possibly does not exist in the current data stream.

In this paper, the weakest first method was used because it removes the worst-performing expert. But in the weakest first method, the maximum limit of experts in ensemble *K* must be decided carefully in conjunction with constant *γ*; it is possible that new experts could be pruned before they are given a chance to learn.

## Experimental Setup

### Datasets

Four datasets were used for the experiment to check the model’s performance. The datasets are reviews from three websites and available at https://archive.ics.uci.edu/ml/datasets/Sentiment+Labelled+Sentences by Kotzias et al. (2015). The first one is the text reviews of movies from the IMDb website, the second one is the text reviews of products from Amazon, and the last one is the text reviews of restaurants from the Yelp website. Each one of these three datasets contain 1000 text reviews. A fourth dataset was prepared by mixing the text reviews from all three datasets and contain 1500 reviews. Each review in the dataset was labeled as 0 and 1. Label 0 represents the negative, and 1 represents the positive sentiment of the review. The task was to determine the sentiment of each review in the stream. The datasets were preprocessed using natural language processing techniques and converted into a document term matrix. The data stream was created using these document term matrices. All these datasets did not have any known drift. It was fascinating to see how the LFR drift detection algorithm performed on these datasets and how the DAE model handled the false-positive drift detected by the algorithm.

### Algorithms

This experiment designed an ensemble based on the Drift detection algorithm. LFR used for drift detection, and for an ensemble, three base classifiers, Hoeffding Tree, Naïve Bayes, and K-nearest neighbor were used. The results were compared with LFR, which used a single classifier for classification. The proposed model was compared with the five state-of-the-art algorithms. These algorithms are OzaBaggingADWINClassifier (OZA), Accuracy Weighted Ensemble (AWE), Additive Expert Ensemble (AEE), Streaming Random Patches (SRP), and Adaptive Random Forest (ARF) classifier.

### Result measures

The result was evaluated by four matrices: accuracy, recall, precision, and F1-measure. These four measures can explain the model’s performance in the best possible way.

## Results

**Figure 2:**
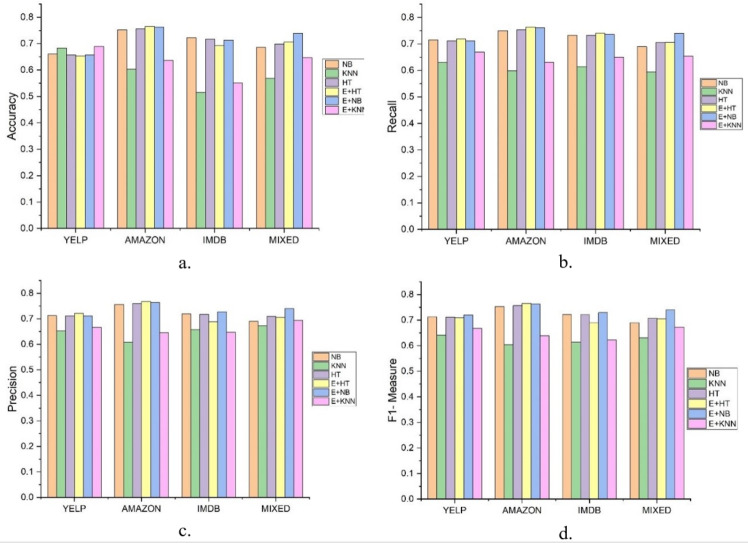
Results of Six classification Models on Different Datasets. The figure compare the six classification models on different parameters. (A) Accuracy, (B) Recall, (C) Precision, (D) F1-measure.

In this experiment, the results were compared between a single classification model with incremental learning and the DAE model, using LFR for drift detection. Hoeffding Tree, K-nearest neighbor (KNN), and Naïve Bayes were used for classification. In Fig. 2, LFR_NB, LFR_KNN, and LFR_HT represent Naïve Bayes, K-nearest neighbor, and Hoeffding Tree classification models with the LFR drift detection algorithm. *DAE_HT*, *DAE_NB*, and *DAE_KNN* represent the DAE using Hoeffding Tree, Naïve Bayes, and KNN as base classifiers. The DAE model also uses LFR for drift detection. So, six classification models were tested on four data streams, and the results are compared in Fig. 2. The first thing we wanted to know is if the DAE performed better than a single classifier model. We concluded this based on four parameters, accuracy, recall, precision, and F1-measure. These six classification models’ accuracies on all four datasets are shown in Fig. 2A.

The accuracy metric shows how accurate classification has been done on stream data. This graph shows that the ensemble with the KNN model did better for the Yelp dataset. The ensemble with the Hoeffding Tree model did better for Amazon, the ensemble with Naïve Bayes did better for the IMDb dataset. For the mixed dataset, the ensemble with Naïve Bayes performed better. But accuracy measures have their limitations as they cannot give a clear picture of the skewed data set. So, we had other performance measures and evaluated each model on these, and the graphs are shown. Fig. 2B shows the value of recall parameters occurring during the experiments. The recall shows how many positive instances were predicted positive. This graph clearly shows that the ensemble classifier gave a better performance and offered a true positive value more than a single classifier. The recall is a necessary parameter when the dataset is skewed, and the number of positive class instances is far less than negative class instances. It shows how classifiers perform for positive class instances. In this experiment, when we mixed the dataset, single classifiers’ recall was lower when compared with the classifiers’ ensemble.

The precision checked how many instances are actually positive for the ones predicted as positive. This shows how precise our model is. In this experiment, the results show that the classifier’s ensemble with each dataset produced better results than the single classifier. Precision gave details on how many positive predicted instances are positive. The ensemble with Naïve Bayes and Hoeffding tree performed better on the Amazon and IMDb datasets on this metric.

F1-measure is the harmonic mean of recall and precision. Higher precision with lower recall or higher recall and lower precision is not good to consider and does not tell the complete story of any model’s prediction capabilities. F1-measures give a better understanding of both the parameters. Figure 2D shows that the value of the F1-measures on any database is better with ensemble than the single classifier model. These experiments prove that the ensemble classifier model gives us a promising solution for data stream analysis.

**Figure 3:**
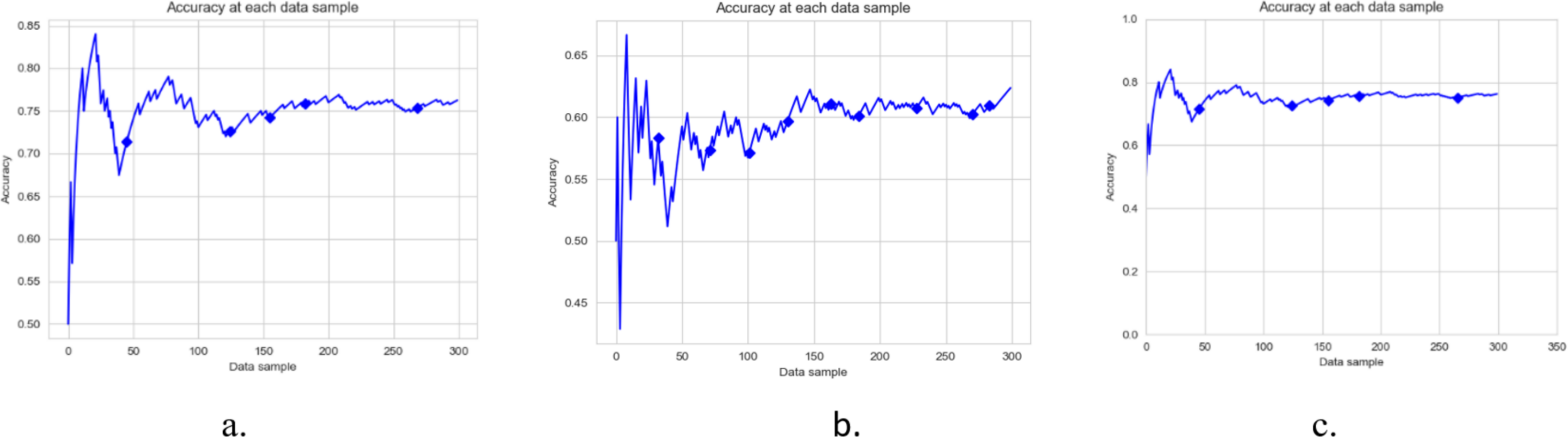
Accuracy of DAE model on Amazon Dataset using different base classifier. Each graph shows the accuracy of DAE model with different base classifiers on the Amazon dataset. The Blue squares show the drift detection point. (A) Hoeffding Tree. (B) KNN, (C) Naive Bayes.

**Figure 4:**
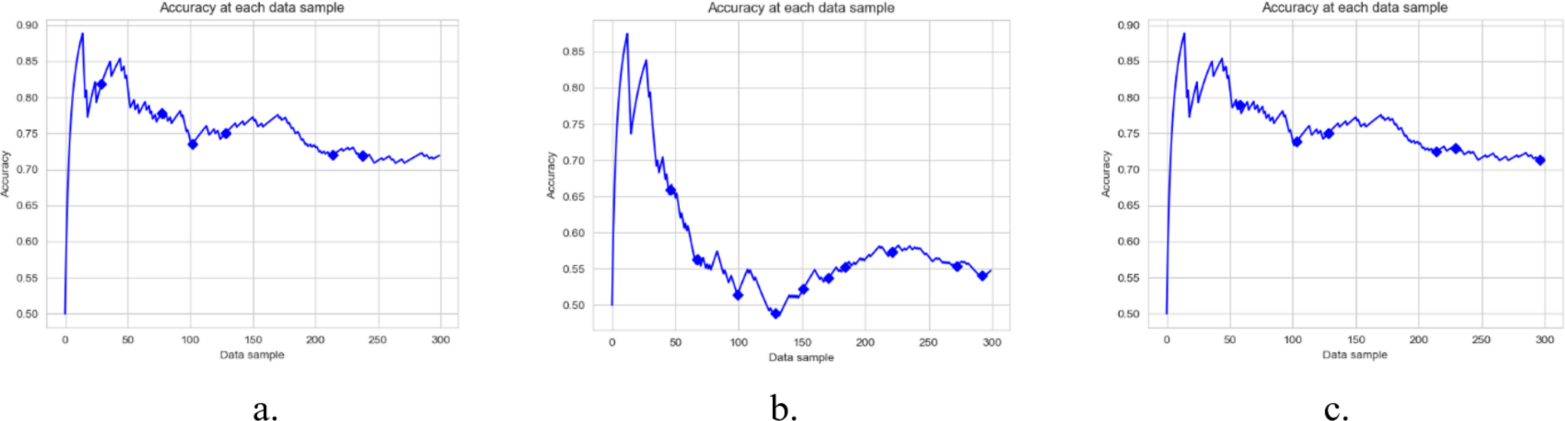
Accuracy of DAE model on IMDB Dataset using a different base classifier. Each graph shows the accuracy of DAE model with different base classifiers on the IMDB dataset. The Blue squares show the drift detection point. (A) Hoeffding Tree. (B) KNN, (C) Naive Bayes.

**Figure 5:**
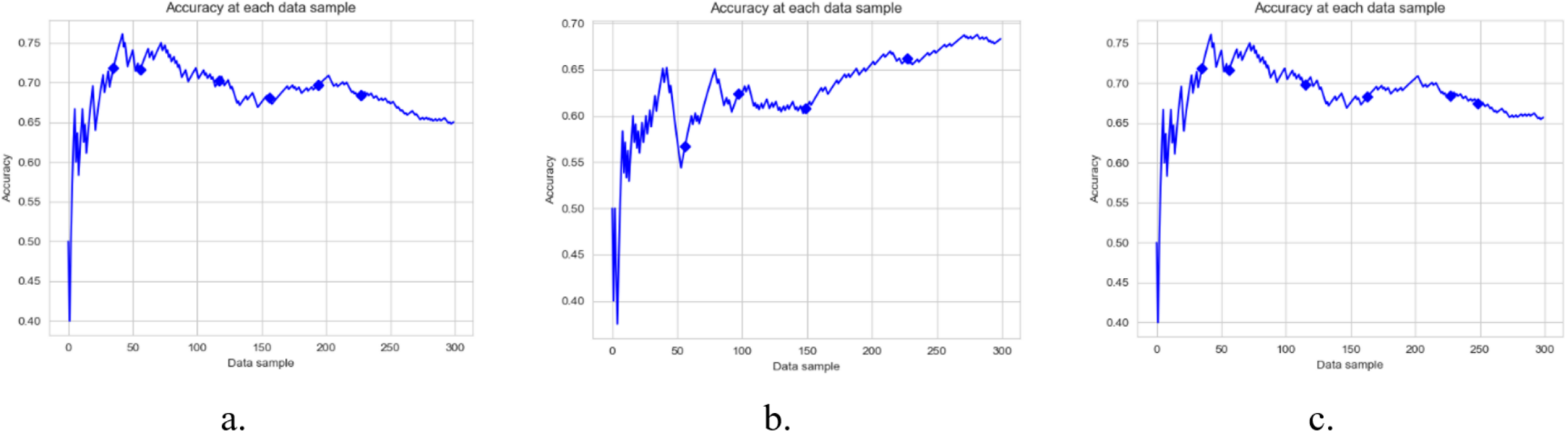
Accuracy of DAE model on YELP Dataset using a different base classifier. Each graph shows the accuracy of DAE model with different base classifiers on the YELP dataset. The Blue squares show the drift detection point. (A) Hoeffding Tree. (B) KNN, (C) Naive Bayes.

**Figure 6:**
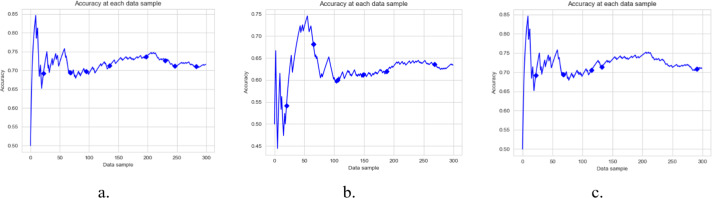
Accuracy of DAE model on Mixed Dataset using a different base classifier. Each graph shows the accuracy of DAE model with different base classifiers on the Mixed dataset. The Blue squares show the drift detection point. (A) Hoeffding Tree. (B) KNN, (C) Naive Bayes.

Figures 3, 4 and 5, and 6 are the DAE model’s accuracy graphs with different base classifiers on all four datasets. These graphs show the drift detected during prediction and how it reflects the classification model’s performance. Figure 3A shows the DAE model’s accuracy with the Hoeffding tree implemented on the Amazon review data set. This graph shows five drifts detected on the stream of 300 data samples. A new classifier was trained and added to the ensemble with each drift. The DAE classifier’s performance was boosted with the increase in the number of classifiers in the ensemble.

**Figure 7:**
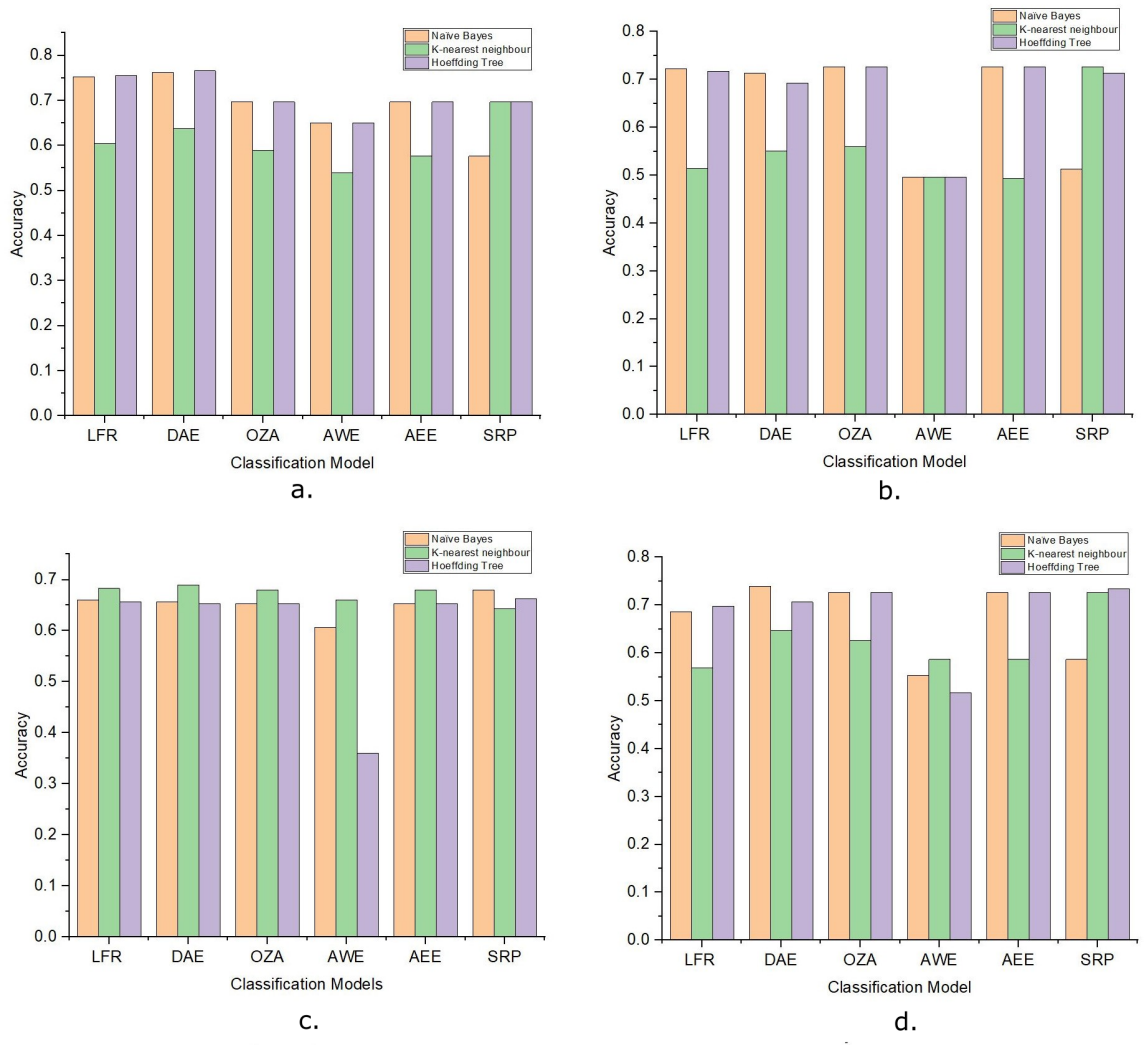
The accuracy of ensemble learners with Naive Bayes, KNN, and Hoeffding Tree as base classifier. (A) Amazon Dataset. (B) IMDb Dataset. (C) Yelp Dataset. (D) Mixed Dataset.

**Figure 8:**
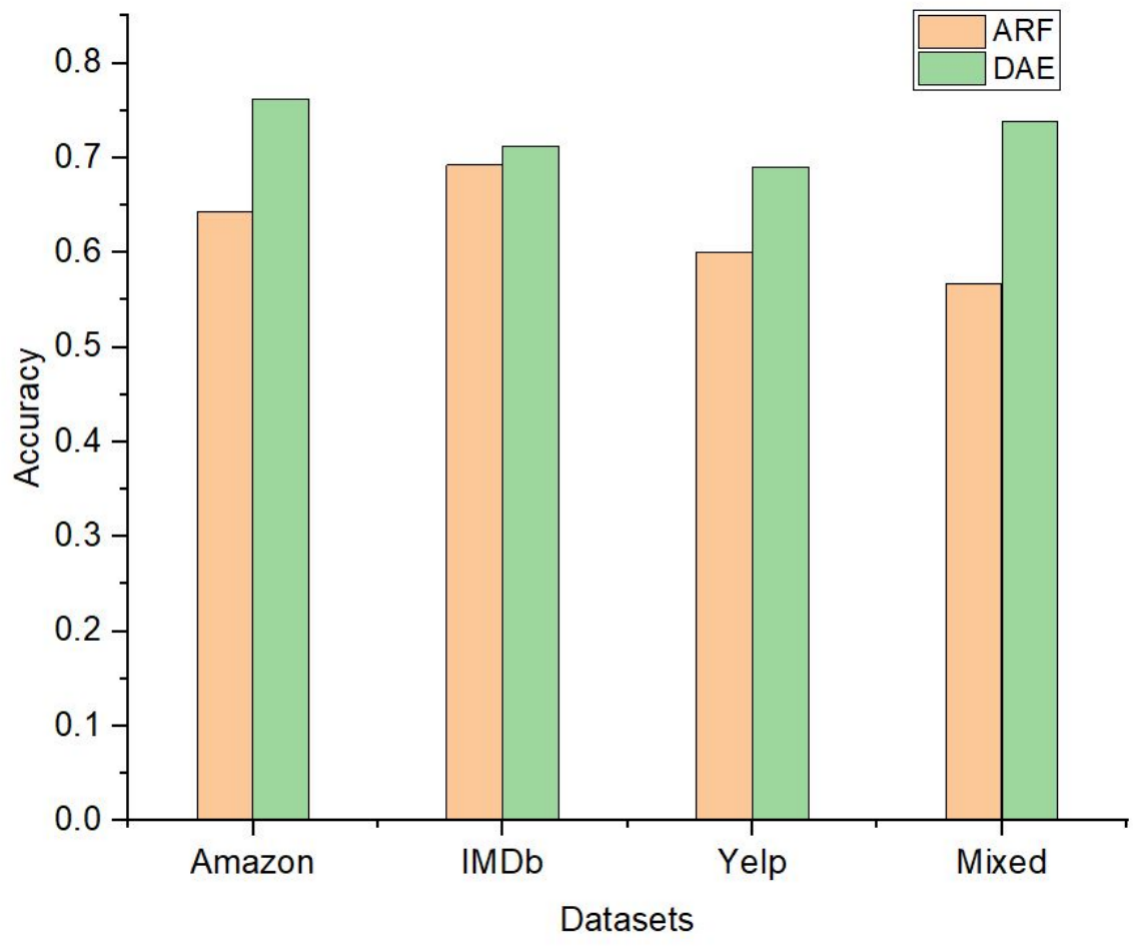
The accuracy comparison between DAE model and ARF classifier.

The performance of the DAE model was compared with five state-of-the-art ensemble classification models. These algorithms were OZA, AWE, AEE, SRP, and ARF. The four algorithms OZA, AWE, AEE, and SRP were implemented with Naïve Bayes, KNN, and Hoeffding Tree base classifiers on four datasets. The results were evaluated and compared with the LFR algorithm and proposed DAE model in Fig. 7. The results of DAE model and ARF are compared in Fig. 8.

Drift detection-based additive ensemble showed substantial results. The DAE performed better on the Amazon dataset than all other algorithms with all three base classifiers. The IMDb dataset OZA and AEE performed with the same accuracy and produced almost the same performance with minor differences. On the Yelp and mixed datasets, the DAE performed better than other classifiers. SRP performed good on IMDb and mixed datasets, but DAE performed better than SRP. In comparing the base classifiers results, KNN was not very effective compared with Naïve Bayes and Hoeffding Tree.

To determine the significance of the evaluated results, one way ANOVA statistical test is performed. Table 3 shows the *p*-values, F-values, and *F*_crit_ after comparing the accuracy score attained by the DAE model and other classifiers. By comparing the DAE model with other state-of-the-art classifiers, the *p*-values obtained are smaller than 0.05, rejecting the null hypothesis that the mean of all the classifiers accuracy score is equal to each other. The F-values obtained using one-way ANOVA test are greater than *F*_crit_ for each combination of DAE model with other classifier. The F-value falls into *F*_crit_ region, which also shows the rejection of null hypothesis.

**Table 3:** The results of one-way ANOVA test between DAE and other state-of-the-art ensemble classifiers.

** Classification Models**	**p-value**	**F-critical**	**F-value**
**DAE *vs* LFR**	0.012640426	2.772853153	4.01923535
**DAE *vs* OZA**	0.018927904	2.772853153	3.6370921
**DAE *vs* AWE**	0.003654049	2.772853153	5.29304821
**DAE *vs* AEE**	0.009455372	2.772853153	4.30330614
**DAE *vs* SRP**	0.009386744	2.772853153	4.31053791

The experiment was repeated on various detect significance levels to determine how the number of drifts detected changes the model’s performance. The Naïve Bayes was used as a base classifier, and the Amazon dataset was used for this experiment. The results are shown in Table 4.

**Table 4:** Accuracy produced by DAE model on Amazon dataset when a base classifier is Naive Bayes with various warning significance levels and detect significance levels for LFR.

**Warning significance level (** *δ* **)**	**Detect significance level (** *ϵ* **)**	**# drift-detected**	**Classification accuracy (%)**
0.3	0.010	2	75.5852843
0.3	0.200	12	75.9197324
0.3	0.040	3	75.5852843
0.3	0.009	2	75.5852843
0.3	0.090	5	76.5886288
0.3	0.150	8	75.5852843

The results show how the change in detecting significance level changes the number of drifts detected during classification. It is possible to control the number of false-positive drift detection using these parameters. Table 4 shows that the accuracy doesn’t decrease if the number of false-positive drift detection increases. In this experiment, the best classification accuracy was achieved when the warning significance level was 0.3, and the detect significance level was 0.09, which resulted in five drifts detected. With these values, the classification accuracy was greater than 76.5%. Figure 7D shows that this is the highest accuracy gain by any other model on the Amazon dataset.

## Discussion

This paper proposes a DAE model for data stream mining with drift detection and adaptation. The experiments were designed to evaluate the performance of the DAE model in such an environment, where it was impossible to determine whether the detected drift is true or false. The DAE model proposed a solution for false-positive drift detection. False-positive drift detection degraded the classification model’s performance. The DAE model created an ensemble classifier that gained strength with each detected drift without knowing if the drift was true or not. After experiments, the results were compared between the DAE model and LFR in Fig. 2. The results show that the DAE model performed better than LFR. The results were evaluated and compared on four metrics, accuracy, recall, precision, and F1-measure. The DAE model showed better results on all four metrics. The accuracy graph in Figs. 3 and 4, 5, and 6 shows the change in accuracy with drift detection points. Multiple drifts were detected in the dataset, and with each drift, the performance of the DAE model remained stable. Figure 3A shows the accuracy of the DAE model with the Hoeffding Tree base classifier. A new classifier was added to the ensemble with each drift, and the DAE model’s accuracy further improved and became stable. The same happens with the other accuracy graphs. This shows the effectiveness of the DAE model. The DAE model was compared with five popular ensemble classifier methods for data stream mining.

OZA is another ensemble classifier that uses a drift detection algorithm. OZA uses ADWIN for drift detection and Bagging as a classification model. AWE and AEE are also two popular ensemble methods for data stream mining, but these methods do not use any drift detection algorithm for drift detection. SRP combines random subspace and online bagging and used ADWIN for drift detection. The comparative results are shown in Fig. 7 for all four datasets. The DAE performed better than all four ensemble methods on the Amazon dataset. The performance of the DAE model on the mixed dataset was also better than other ensemble classifiers. The DAE model performed like different ensemble classifiers on IMDb and Yelp datasets. The one-way ANOVA test is done between DAE model and state-of-the-art ensemble classifiers. The *p*-value is less than 0.05 in each case, which rejects the null hypothesis and shows that the results are very significant. These results show that the DAE is very effective, even if the drift detection algorithm signals multiple false-positive drifts. Table 4 shows the number of drift and the accuracy of the DAE model on the Amazon dataset with multiple detect significance level values. The number of drifts detected changes with the change in detect significance levels, but the classification model’s accuracy did not degrade and remained stable. The LFR drift detection algorithm minimizes the number of false-negative drift detection, and because of this, the number of false-positive detections increases. The DAE model could handle and adapt the true drift and the false-positive drift. The performance of the classification model improved and remained stable with each drift. From all these results, it is concluded that the DAE model is an effective model for data stream mining.

## Conclusions

This paper introduces a technique to design an adaptive ensemble classifier for online sentiment analysis and opinion mining. Online sentiment analysis is a demanding task because of the greater dimensionality and concept drift in the text data stream. False-positive drift detection by any drift detection algorithm negatively affects the classification performance. The proposed adaptive ensemble classifier provided an approach to reduce the effect of false-positive drift detection and efficiently deal with the true positive drift. In this work, LFR was applied for drift detection due to its effectiveness with drift for balanced and imbalanced datasets. LFR uses four parameters for drift detection, which are extremely sensitive to any changes in data distribution and are prone to false-positive drift detection. The proposed method generated and included a classification model to the ensemble whenever a drift was encountered.

The newly added classification model was trained on recent datasets. If the detected drift was a false positive drift, then the newly added classifier would strengthen the ensemble. If the detected drift was a true positive, the newly added classifier would lead the classification results because of the higher weightage given to this model. The proposed method was implemented with three different classification models as the base classifier: Hoeffding Tree, Naïve Bayes, and KNN. The experiments were executed on four datasets, and results were evaluated. The results of this adaptive ensemble classifier were compared with single classification models. The results show that adaptive ensemble classifiers had better results than single classification models. The proposed model was also compared with five state-of-the-art classification models for stream analysis: AEE, AWE, OZA, SRP, and ARF. The results show that the proposed model provided better accuracy on three out of four datasets. The results are promising, and the proposed model has a significant contribution to present knowledge of text data stream mining. In this work, the drift detection algorithm LFR took longer than expected to work on a higher-dimensional text dataset. Because of this, the experiments were conducted on a restricted volume of data. This limitation needs to be eliminated in future work, so the proposed model becomes faster and more robust. Other than that, different weightage mechanisms and pruning mechanisms can increase robustness to work on any datasets. The dataset used did not have any known drift, so it is not possible to say that the drifts that were found were legitimate or not. But we can understand that the proposed model performed better than other classifiers, which explains that the proposed model is truly effective and can be applied in future research works.

##  Supplemental Information

10.7717/peerj-cs.660/supp-1 Supplemental Information 1 Python code for proposed DAE model

10.7717/peerj-cs.660/supp-2 Supplemental Information 2 The text based review dataset taken from Amazon, Imdb, and Yelp websites

## Additional Information and Declarations

### Data Availability

The following information was supplied regarding data availability: The raw review data is available in the Supplemental Files.
